# Effect of Naoxintong Capsules on the Activities of CYP450 and Metabolism of Metoprolol Tartrate in Rats Evaluated by Probe Cocktail and Pharmacokinetic Methods

**DOI:** 10.1155/2019/5242605

**Published:** 2019-09-23

**Authors:** Huizi Ouyang, Jiayuan Shen, Xuhua Huang, Wenjuan Ma, Qi Jia, Guangzhe Yao, Zhidan Tang, Dandan Zhang, Mengjie Sun, John Teye Azietaku, Jia Hao, Xiumei Gao, Yanxu Chang, Jun He

**Affiliations:** ^1^First Teaching Hospital of Tianjin University of Traditional Chinese Medicine, Tianjin 300193, China; ^2^Tianjin State Key Laboratory of Modern Chinese Medicine, Tianjin University of Traditional Chinese Medicine, Tianjin 301617, China

## Abstract

Naoxintong capsule (NXT), a prescribed Chinese medicine, has been used clinically for more than 20 years and is widely received by patients. We determined five probe drugs, namely, omeprazole (CYP2C19), midazolam (CYP3A4), phenacetin (CYP1A2), tolbutamide (CYP2C9), and dextromethorphan (CYP2D6) to study the potential influences of NXT on the activities of CYP enzymes and assessed the pharmacokinetics effect of NXT on metoprolol tartrate in rat plasma. The study showed that AUC_(0–24)_ and AUC_(0–∞)_ of midazolam (CYP3A4) in NXT coadministration group (283.7 ± 65.2 h·ng·mL^−1^ and 292.0 ± 75.1 h·ng·mL^−1^ in group B; 295.7 ± 62.7 h·ng·mL^−1^ and 299.5 ± 60.0 h·ng·mL^−1^ in group C) were significantly decreased as compared to another group (416.8 ± 82.3 h·ng·mL^−1^ and 424.9 ± 77.9 h·ng·mL^−1^ in group A), while that of dextromethorphan (CYP2D6) showed an opposite tendency (540.7 ± 119.7 h·ng·mL^−1^ and 595.3 ± 122.2 h·ng·mL^−1^ in group A, 760.6 ± 184.9 h·ng·mL^−1^ and 788.7 ± 211.0 h·ng·mL^−1^ in group B, and 734.3 ± 118.5 h·ng·mL^−1^ and 757.2 ± 105.4 h·ng·mL^−1^ in group C). Moreover, NXT preadministration can enhance the metabolism of metoprolol tartrate and reduce the metabolism of O-demethylmetoprolol. The results indicated that NXT had potential effects in inducing CYP3A4 and inhibiting CYP2D6 in the metabolism of metoprolol tartrate. It suggests that patients who coadministered NXT and metoprolol tartrate should be advised of potential herb-drug interactions (HDIs) to reduce therapeutic failure or accelerated toxicity of conventional drug treatment.

## 1. Introduction

Chinese medicinal herbs (CMHs) have been used for cerebrovascular disease clinically because of its fewer side effects and multitargeted effects [1]. NXT is a famous Chinese medicine for the treatment of cardiovascular diseases in clinic for more than 20 years [2, 3] and is composed of 16 CMHs including *Radix Angelicae Sinensis* (Danggui), *Commiphora myrrha Eng1* (Moyao), *Radix Paeoniae* (Chishao), *Semen Persicae* (Taoren), *Salviae miltiorrhizae Radix et Rhizoma* (Danshen), and *Achyranthes bidentata* (Niuxi) [4]. This prescription has multifaceted pharmacological activities, such as advance tissue regeneration, inhibition of dendritic cell maturation, improvement in mitochondrial membrane potential, reduction in lipid concentrations, inhibition of platelet aggregation, and anti-inflammatory and antioxidative properties [5, 6]. NXT has been widely used in treating atherosclerosis, cerebrovascular diseases, blood stasis syndrome, qi deficiency, and coronary heart diseases [7]. Furthermore, it has increased popularity as a coadministered treatment with other prescription drugs. For example, NXT combined with dual antiplatelet therapy (clopidogrel and aspirin) may reduce the risk of intraoperative bleeding, decrease coronary microembolization, and inhibit platelet aggregation [8]. NXT combined with metoprolol tartrate has been used to treat cardio-cerebrovascular diseases and improve patient's blood lipid metabolism [9]. Increasing publications have reported that the regulation of CYP450 enzymes is a pivotal cause of adverse herb-drug interactions (HDIs) [10]. Chen [11, 12] found that NXT could significantly promote the catalytic activity of CYP2C19, while further studies showed that the combined use of NXT and clopidogrel exhibited increased antiplatelet effect in patients with CYP2C19 *∗* 2 gene mutation after percutaneous coronary intervention (PCI) and reduced secondary angiotensinoma.

**Figure 1:**
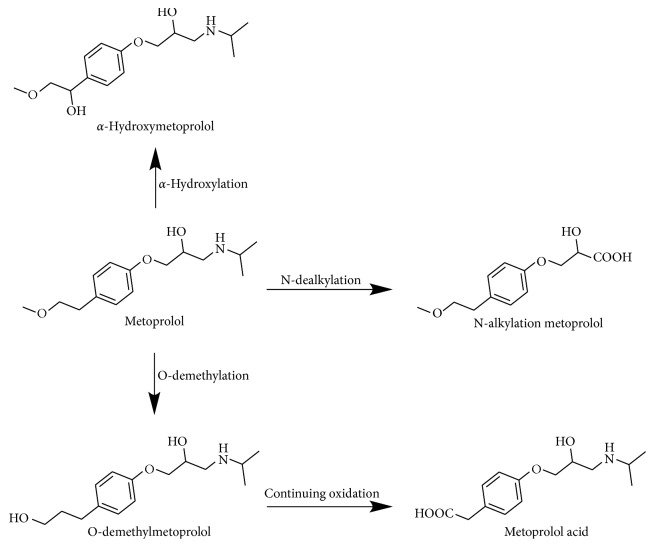
The metabolic pathways of metoprolol tartrate.

Metoprolol is a cardioselective beta-blocker used in the treatment of hypertension, angina pectoris, coronary heart diseases, and myocardial infarction [13, 14]. It could also be used during pregnancy owing to its low fetal and neonatal risk [15]. Studies have reported that metoprolol is mainly metabolized by cytochrome and is almost exclusively eliminated by hepatic metabolism [16]. The major oxidative pathways of metoprolol are O-demethylation to O-demethylmetoprolol, which is further oxidized into its corresponding metoprolol phenylacetate (65% of the oral dose recovered in urine), and N-dealkylation to N-diisopropylmetoprolol (10%) [17]. *α*-Hydroxylation, a metabolic component of metoprolol, is catalyzed almost entirely, and O-demethylation of metoprolol is catalyzed partially by human P450 2D6 [18]. The metabolic pathways of metoprolol and its metabolites are shown in Figure 1. Latest research shows that metoprolol is not completely metabolized by CYP2D6. CYP3A4, 2B6, and 2C9 can also promote *α*-hydroxylation, O-demethylation, and N-dealkylation of metoprolol [17].

CYP450 family is the key enzyme system playing a vital role in metabolizing various drugs, xenobiotics, and endogenous substances [19–22], and it is responsible for the metabolism of over 70–80% of the rate-limiting phase I metabolism of drugs [23]. Changes in the activity of CYP450 may influence plasma levels of a concomitantly administered drug, thereby increasing the incidence of drug-induced toxicity, causing HDIs clinically [24, 25]. Such problems have been reported in the last few years and have aroused wide public attention with respect to clinical drug safety [26]. Evaluation of potential effects of Chinese medicines on CYP450 enzyme activities, especially for drugs with narrow therapeutic index, is essential and important in predicting HDIs and toxic effects [27]. Cocktail approach, a method widely used to assess the potential effects of drugs on CYP450 enzyme activities, has been developed and evaluated for the different impacts of a drug on these probe drugs metabolized by CYP450. In addition, this method is also used to study HDIs [28]. The issue of HDIs has caused wide public concern since CMHs were extensively used in the world. Clinically, the coadministration of NXT and metoprolol tartrate has been effectively used for treating unstable angina pectoris [9], but potential adverse action of HDIs remains to be verified. Therefore, we should pay more attention to the impacts of HDIs when NXT is combined with other pharmaceutical drugs. The aim of this research is to evaluate the influence of NXT on CYP450 enzymes and to investigate the impact of NXT on the metabolism of metoprolol tartrate. The results would be useful for clinical safety evaluation of HDIs involving NXT.

## 2. Materials and Methods

### 2.1. Chemicals, Reagents, and Materials

Acetonitrile and methanol of HPLC grade were supplied by Merck. Formic acid of HPLC grade was obtained from ROE. Acetic acid of chromatographic purity was purchased from TEDIA Co. Ltd. Ultrapure water was purified through a Milli-Q system. Omeprazole (99.9%, batch number: 100367–201305) and dextromethorphan (95.1%, batch number: 100201–201003) were purchased from China National Institutes for Food and Drug Control. Midazolam (99.8%, batch number: 20121213) was obtained from Shanghai Neuen Biology Technology Co. Ltd. Phenacetin (≥99%, batch number: 06 A 13) and tolbutamide (≥99%, batch number: 12 A 12) were purchased from Tianjin Semar Technology Co. Ltd. Diazepam (≥98%, batch number: H32020730) and propranolol hydrochloride (batch number: 100783–101202) as internal standards were obtained from Tianjin Silan Technology Co. Ltd and Chinese Food and Drug Test Institute, respectively. Metoprolol tartrate (batch number: 100084–201403) was purchased from Chinese Food and Drug Test Institute. O-demethylmetoprolol (batch number: 100084–201403) and *α*-hydroxymetoprolol (batch number: 1356–031A6) were obtained from Canada. Naoxintong capsule (batch number: 1608102) was supplied by Shanxi Buchang Pharmaceutical Co. Ltd.

### 2.2. Animals and Treatment

Male Sprague Dawley rats (200 ± 20 g) were used for pharmacokinetic analysis. The rats were raised with standard diet and water in a stable environment, with temperatures between 23 and 26°C and relative humidity of 40–60%. After a week of acclimatization, the rats were fasted 12 h before PK experiment and allowed free access to water during the experiment.

The rats were randomly divided into CYP probe groups and metoprolol tartrate group. According to the recent study [29–31], we formulated the dose and route of administration of probe substrates. The CYP probe group comprised of three subgroups: rats in group A were given 2.5 mL/kg solution of five probe drugs (5 mg/kg of omeprazole and phenacetin, 2 mg/kg of midazolam and dextromethorphan, and 4 mg/kg of tolbutamide) through tail vein; rats in group B were administered 500 mg/kg NXT by oral administration and then after 5 minutes, dosed with 2.5 mL/kg solution of five probe drugs through tail vein; rats in group C were given 500 mg/kg NXT by oral administration daily for 8 consecutive days and dosed through tail vein with a solution of five probe drugs on the eighth day.

Rats of metoprolol tartrate study also comprised of three subgroups: rats in group A′ were given 20.8 mg/kg solution of metoprolol tartrate; rats in group B′ were given metoprolol tartrate solution orally at 20.8 mg/kg and NXT solution orally at 500 mg/kg at single time dose; rats in group C′ were given 500 mg/kg NXT solution for 7 consecutive days and orally administrated metoprolol tartrate solution at 20.8 mg/kg and NXT solution at 500 mg/kg on the eighth day.

The animal studies described in this paper were approved and conducted in accordance with the guidelines of the Laboratory Animal Ethics Committee of Tianjin University of Traditional Chinese Medicine (TCM-LAEC20170054).

### 2.3. Sample Preparation

#### 2.3.1. CYP Probe Drug Groups

Blood samples of each rat were collected at time points of 0.08, 0.17, 0.25, 0.33, 0.50, 0.75, 1, 2, 4, 6, 8, 12, and 24 h from the fossa orbitalis vein after administration. Blood samples (200 *μ*L) were transferred into a heparinized microcentrifuge tube and centrifuged at 6000 rpm for 10 min. All plasma samples were stored at –70°C until use.

100 *μ*L plasma sample, 25 *μ*L diazepam (internal standard solution, 1000 ng/mL), and 25 *μ*L methanol were added into a centrifuge tube and mixed by vortexing. Liquid-liquid extraction was performed by adding 1 mL ethyl acetate into the centrifuge tube. The mixture was vortexed for 3 min and centrifuged at 14,000 rpm for 10 min. 900 *μ*L supernatant was transferred into a new tube and evaporated to dryness. The residue was reconstituted with 100 *μ*L methanol. The solution was then vortexed for 3 min and centrifuged at 14,000 rpm for 10 min. 5 *μ*L supernatant was injected into the LC-MS/MS system.

#### 2.3.2. Metoprolol Tartrate Groups

Blood samples were collected in Eppendorf tubes coated with heparin sodium from rats after oral administration at 0, 0.03, 0.08, 0.17, 0.25, 0.5, 1, 2, 4, 8, 12, and 24 h, following which the blood samples were immediately centrifuged at 6000 rpm for 10 min. These plasma samples were stored at −70°C until use.

The plasma (100 *μ*L) was spiked with 25 *μ*L of methanol and 25 *μ*L of IS (propranolol hydrochloride, 800 ng/mL). After vortex mixing, 800 *μ*L of ethyl acetate was added and then vortex-mixed for 3 min at room temperature. After centrifugation at 14,000 rpm for 5 min, the supernatant was collected to a clean tube and evaporated to dryness under a nitrogen stream. The residue was reconstituted in 100 *μ*L of 50% methanol (v/v), vortexed for 5 min, and centrifuged at 14,000 rpm for 10 min. Appropriate amounts of supernatant were injected into the LC-MS/MS system for analysis.

### 2.4. Analytical Methods

#### 2.4.1. CYP Probe Groups

Chromatographic separation was performed using Agilent 1200 HPLC system, and the analysis was achieved on Waters XBridgeTM C18 column (2.1 × 100 mm, 3.5 *μ*m) with temperature maintained at 30°C. Gradient elution was carried out with mobile phase A (0.1% formic acid in water) and mobile phase B (acetonitrile) as follows: 0–6.0 min, 25–80% B; 6.0–7.0 min, 80% B. The flow rate was 0.4 mL/min and the injection volume was 5 *μ*L.

MS spectrometer detection was operated on an Agilent 6430 triple quadrupole mass spectrometer coupled with an electrospray ionization (ESI) source. The mass spectrometer was set in positive ion mode. The optimum MS values were maintained as follows: nebulizer, 25 psig; drying gas (N_2_) flow rate at 11 L/min; and capillary temperature at 300°C. The precursor-to-product ion pairs, fragmentor, and collision energy (CE) for five probe drugs and IS are shown in Table 1.

#### 2.4.2. Metoprolol Tartrate Groups

The analysis conditions, which include HPLC system, column, and temperature, were the same as described for that of CYP probe drug groups. Mobile phases of metoprolol tartrate, O-demethylmetoprolol, *α*-hydroxymetoprolol, and propranolol hydrochloride (IS) consisting of 0.05% acetic acid in water (A) and acetonitrile (B) were used in the following gradient elution method: 0–4 min, 10%–90% B; 4–6 min, 90%–10% B.

MS spectrometer detection was operated on a triple quadrupole mass spectrometer coupled with an electrospray ionization (ESI) source. The mass spectrometer was also set in positive ion mode. The source parameters of metoprolol tartrate, O-demethylmetoprolol, *α*-hydroxymetoprolol, and propranolol hydrochloride (IS) were as follows: nebulizer, 30 psig; drying gas (N_2_) flow rate at 13 L/min; and capillary temperature at 305°C. The precursor-to-product ion pairs, fragmentor, and collision energy (CE) for the analytes are shown in Table 2.

### 2.5. Statistical Analysis

All data acquisition and peak integration were performed using Analyst software (versions B.04.00) from Agilent MassHunter. Pharmacokinetic parameters were calculated using the computer program “Drug and Statistics 1.0” (DAS 1.0; Medical College of Wannan, China). Statistical comparisons were made by one-way-ANOVA in SPSS l7.0 statistical software.

## 3. Results

### 3.1. Effect of NXT on Five Probe Drugs in Rats

**Figure 2:**
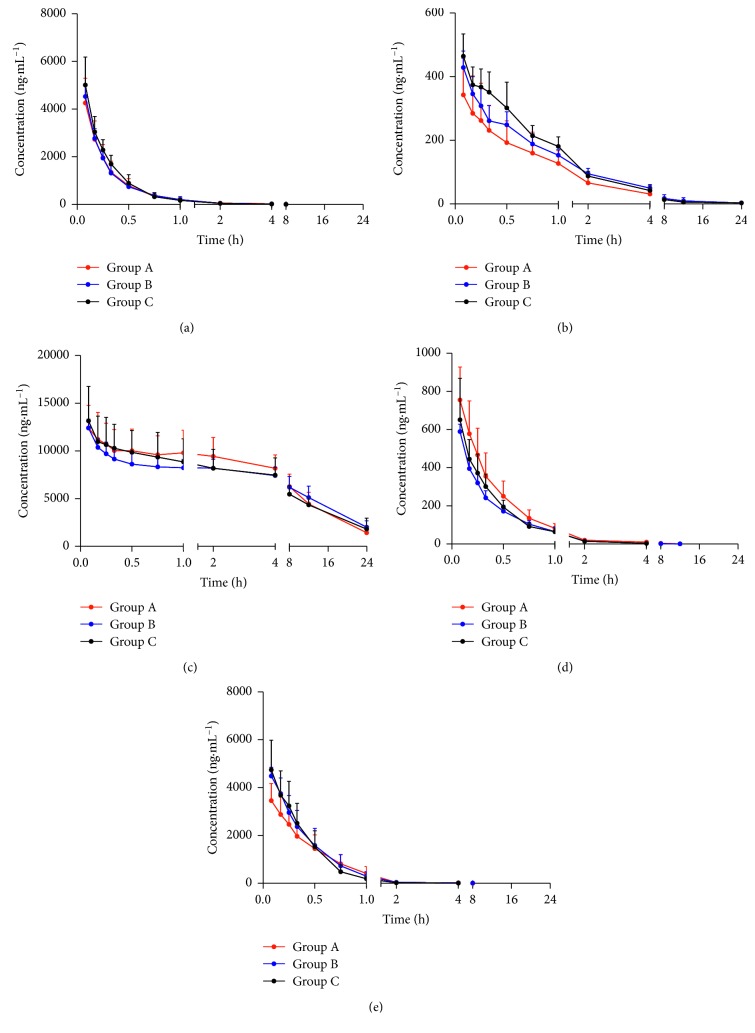
Mean plasma concentration-time curves of probe drugs in different groups (*n* = 6). Group A received 2.5 mL/kg solution of five probe drugs through the tail vein; group B received 500 mg/kg Naoxintong capsules by oral administration and then dosed 2.5 mL/kg solution of five probe drugs through the tail vein; group C received 500 mg/kg Naoxintong capsules by oral administration daily for 8 consecutive days and dosed through the tail vein with 2.5 mL/kg solution of five probe drugs on the eighth day. (a) Omeprazole, (b) dextromethorphan, (c) tolbutamide, (d) midazolam, and (e) phenacetin.

This experiment studied the effect of NXT on omeprazole (CYP2C19), midazolam (CYP3A4), phenacetin (CYP1A2), tolbutamide (CYP2C9), and dextromethorphan (CYP2D6) in different treatment groups as mentioned in Section 2.2. The pharmacokinetic parameters and mean plasma concentration-time curves of the five probe drugs are shown in Table 3 and Figure 2.

#### 3.1.1. Effect of NXT on Omeprazole (CYP2C19) in Rats

The effects of NXT in the different treatment groups on pharmacokinetic parameters including *C*_max_ (4256.2 ±1043.3 ng·mL^−1^ in group A, 4527.9 ± 534.1 ng·mL^−1^ in group B, and 5010.3 ± 1175.5 ng·mL^−1^ in group C), AUC_(0–24)_ (1483.9 ±366.0 h·ng·mL^−1^ in group A, 1459.9 ± 401.0 h·ng·mL^−1^ in group B, and 1601.7 ± 416.6 h·ng·mL^−1^ in group C) and AUC_(0–∞)_ (1534.2 ± 383.7 h·ng·mL^−1^ in group A, 1485.3 ± 407.4 h·ng·mL^−1^ in group B, and 1665.8 ± 513.9 h·ng·mL^−1^ in group C) of omeprazole in rat plasma and the mean plasma concentration-time curves of omeprazole are presented in Table 3 and Figure 2. There were little changes in pharmacokinetic results among the three groups. It was shown that NXT had no influence on CYP2C19 activity in vivo.

#### 3.1.2. Effect of NXT on Midazolam (CYP3A4) in Rats

As shown in the results, the AUC_(0–24)_ and AUC_(0–∞)_ of midazolam in groups B (283.7 ± 65.2 h·ng·mL^−1^ and 292.0 ±75.1 h·ng·mL^−1^) and C (295.7 ± 62.7 h·ng·mL^−1^ and 299.5 ±60.0 h·ng·mL^−1^) were decreased significantly (*P* ≤ 0.01) as compared to group A (416.8 ± 82.3 h·ng·mL^−1^ and 424.9 ±77.9 h·ng·mL^−1^). The results indicated that NXT may have the potential effects to induce CYP3A4 activity in vivo.

#### 3.1.3. Effect of NXT on Phenacetin (CYP1A2) in Rats

Pharmacokinetic profiles of phenacetin after NXT treatment were used to describe the activity of CYP1A2. As shown in Table 3, the AUC_(0–24)_ and AUC_(0–∞)_ of phenacetin in groups A, B, and C were 1886.7 ± 602.0 h·ng·mL^−1^ and 1944.1 ± 578.1 h·ng·mL^−1^, 2025.4 ± 512.1 h·ng·mL^−1^ and 2067.7 ± 509.9 h·ng·mL^−1^, and 1922.8 ± 595.2 h·ng·mL^−1^ and 1943.5 ± 581.4 h·ng·mL^−1^, respectively. There was no significant difference in the pharmacokinetic parameters in the rats among the different groups, indicating that NXT had little impact on CYP1A2 activity in vivo.

#### 3.1.4. Effect of NXT on Tolbutamide (CYP2C9) in Rats

Alteration in CYP2C9 activity induced by NXT was estimated by comparing the pharmacokinetic results of tolbutamide in rats by different treatment methods. As compared to group A (122412.8 ± 25958.5 h·ng·mL^−1^), the AUC_(0–24)_ of tolbutamide in group B (124685.2 ±19824.0h·ng·mL^−1^) and C (112713.7 ± 19708.0 h·ng·mL^−1^) showed that there was no significant influence in the different treatment groups. These results implied that NXT showed little alteration on CYP2C9 activity in vivo.

#### 3.1.5. Effect of NXT on Dextromethorphan (CYP2D6) in Rats

Alteration of CYP2D6 activity induced by NXT was evaluated by monitoring the pharmacokinetics of dextromethorphan. AUC_(0–24)_ of dextromethorphan in groups B (760.6 ± 184.9 h·ng·mL^−1^) and C (734.3 ± 118.5 h·ng·mL^−1^) were increased (*P* ≤ 0.05) as compared to group A (540.7 ± 119.7 h·ng·mL^−1^), indicating that NXT may inhibit the activity of CYP2D6.

### 3.2. Effect of NXT on the Pharmacokinetics of Metoprolol Tartrate

**Figure 3:**
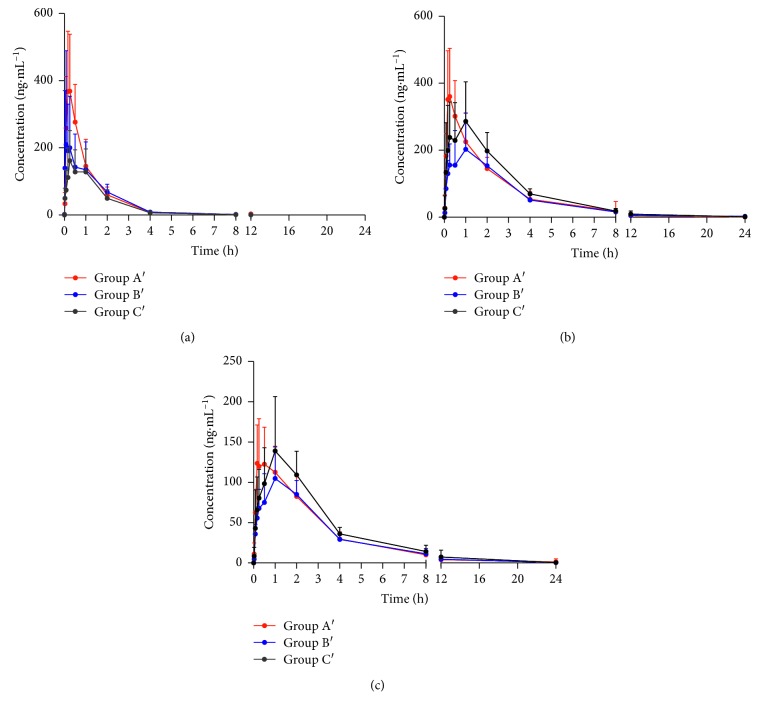
Mean serum concentration-time profiles of metoprolol tartrate and its metabolites (*n* = 6). Group A′: a single oral dose of metoprolol tartrate (20.8 mg/kg), group B′: coadministration of NXT (500 mg/kg) and metoprolol tartrate (20.8 mg/kg), group C′: oral administration of NXT (500 mg/kg) for 7 consecutive days and on the eighth day metoprolol tartrate (20.8 mg/kg) and NXT (500 mg/kg). (a) Metoprolol, (b) *α*-hydroxymetoprolol, and (c) O-demethylmetoprolol.

HPLC-MS/MS method was applied to the pharmacokinetic study of the analytes in the rat blood sample after oral administration according to the different grouping methods. The mean concentration-time curves of metoprolol and its two metabolites in different ways of administration are shown in Figure 3. The major pharmacokinetic parameters are demonstrated in Table 4.

The AUC_(0–tn)_ and AUC_(0–∞)_ of metoprolol in group C′ (280 ± 69 h·ng·mL^−1^, 285 ± 70 h·ng·mL^−1^) were significantly lower (*P* ≤ 0.01) than group A′ (454 ± 113 h·ng·mL^−1^, 529 ± 243 h·ng·mL^−1^), suggesting that NXT had a significant effect on metoprolol in group C′, as compared to the group A′. However, the AUC_(0–tn)_ (456 ± 91 h·ng·mL^−1^ in A′ group and 536 ± 145 h·ng·mL^−1^ in C′ group) and AUC_(0–∞)_ (473 ± 126 h·ng·mL^−1^ in A′ group and 540 ± 146 h·ng·mL^−1^ in C′ group) of O-demethylmetoprolol showed an opposite tendency, indicating that the metabolism of O-demethylmetoprolol was slowed down, probably because NXT could promote CYP450 enzyme activity to catalyze O-demethylation of metoprolol.

In this experiment, we found that the *T*_max_ was delayed for parent and metabolite and the *C*_max_ was decreased for metoprolol; the results were similar to the research of Sun et al. [29] and Hsueha et al. [32, 33]and the *C*_max_, *T*_1/2_, and AUC showed similar tendency in the group of herb-drug combination compared with drug administration alone. The changes of PK parameters for metoprolol might be influenced by the metabolic enzymes. Meanwhile, this phenomenon might also be caused by the absorption of metoprolol in vivo after oral administration of NXT.

The pharmacokinetic results above demonstrated that pretreatment with NXT for eight consecutive days showed statistically significant interaction with metoprolol tartrate and its metabolites in rats.

## 4. Discussion

Herbs have been used for clinical treatment for a long time before the development of modern medicines. It is calculated that about 80% of the Asian population used CMHs as complementary or alternative medicine [34]. However, herb-drug interactions (HDI) might occur during the process of clinical application. The important metabolic enzyme, cytochrome P450, plays a vital role in drug metabolism [35]. CYP450 is a superfamily of enzymes, and the majority of CYPs involved in drug metabolism include CYP3A4/5, CYP2D6, CYP1A2, CYP2B6, CYP2C19, CYP2A6, and CYP2J2. When one of the drugs inhibits CYP enzyme activity, drug-drug interactions may occur, affecting the metabolism of the coadministered drug [35, 36].

NXT has been widely used in the clinical treatment of cerebral infarction and carotid atherosclerosis [37]. It has become increasingly popular to be used in combination with other prescription drugs. Danshen is one of the main components of NXT; its chemical composition tanshinones plays an important role in the inhibition and induction of CYP450 isozymes [38]. Danggui, another component of NXT, also influences the expression of hepatic CYP450 isoforms [39]. These studies drew our attention to research about NXT-mediated pharmacokinetic behaviors alteration of metoprolol tartrate, especially its effects on CYP450.

In this research, rats were chosen as the experimental animal model. Although human's isoform composition, expression, and catalytic activities of drug-metabolizing enzymes are different from those of rats, rats are common animal models for metabolic behavior studies. We investigated the potential effects of NXT on the CYP450 activities in rats, using the probe cocktail method to determine the pharmacokinetic behaviors of five probe drugs—omeprazole (CYP2C19), midazolam (CYP3A4), phenacetin (CYP1A2), tolbutamide (CYP2C9), and dextromethorphan (CYP2D6) respectively. The results suggested that NXT has significant effects on CYP3A4 and CYP2D6, influencing the metabolism of metoprolol tartrate and its metabolites (O-demethylmetoprolol). These results are consistent with previous reports on the metabolism of metoprolol tartrate [17]. Increasing evidence demonstrated that the induction/inhibition of CYP enzymes is one of the risk factors for HDIs. Hence, further studies should be carried out to investigate the influence of NXT on these drugs.

## 5. Conclusion

Our study was conducted to investigate the influences of NXT on the activities of CYP450 and on the pharmacokinetics of metoprolol tartrate in plasma of rats. The results indicated that NXT may induce CYP3A4 activity while inhibiting CYP2D6 activity in vivo, and pretreatment with NXT increased metabolism of metoprolol tartrate and decreased metabolism of O-demethylmetoprolol. The findings from this study have demonstrated that NXT capsule may have the potential to induce CYP3A4 while inhibiting CYP2D6, thus affecting the metabolism of metoprolol tartrate and its metabolites. And this phenomenon might also be caused by the absorption of metoprolol in vivo after oral administration of NXT. Therefore, HDIs should be considered when NXT is used in combination with other drugs, especially those metabolized by CYP3A4 and CYP2D6. Some studies also showed that herb-drug interactions with western medicine are potentially causing changes in drug levels and drug activities, leading to either side effects or toxicities that can sometimes be fatal [40]. The exposure to drugs and lack of knowledge in the potential adverse herb-drug interactions puts big risk to patient safety in medical services. Hence, it is important to have a widespread concern regarding the combined use of TCM and western medicine clinically, so as to prevent HDI-related toxicity and side effects.

## Figures and Tables

**Table 1:** MS parameters of the five probe drugs and IS.

Probe drug	Precursor ion (*m*/*z*)	Product ion (*m*/*z*)	Fragmentor (V)	CE (V)
Omeprazole	346.2	136.1	80	35
Midazolam	326.1	291.1	160	25
Dextromethorphan	272.2	147.1	130	30
Phenacetin	180.1	110.1	100	17
Tolbutamide	271.1	91.1	100	17
Diazepam (IS)	285.1	193.0	130	30

**Table 2:** Mass spectra properties of the target ingredients.

Analyses	Precursor ion (*m*/*z*)	Product ion (*m*/*z*)	Fragmentor (V)	CE (V)
Metoprolol tartrate	268.2	121.1	110	20
*α*-Hydroxymetoprolol	284.2	116.1	120	14
O-demethylmetoprolol	254.2	116.1	110	23
Propranolol hydrochloride (IS)	260.2	155.1	90	23

**Table 3:** Pharmacokinetic parameters of the five probe drugs in different groups (*n* = 6).

Probe drug and group	*C*_max_ (ng/mL)	*T*_1/2_ (h)	AUC_(0–24)_ (h·ng/mL)	AUC_(0–∞)_ (h·ng/mL)	MRT_(0–24)_ (h)	MRT_(0–∞)_ (h)
*Omeprazole*						
Group A	4256.2 ± 1043.3	0.16 ± 0.03	1483.9 ± 366.0	1534.2 ± 383.7	0.86 ± 0.52	1.26 ± 0.77
Group B	4527.9 ± 534.1	0.15 ± 0.04	1459.9 ± 401.0	1485.3 ± 407.4	0.63 ± 0.53	0.74 ± 0.62
Group C	5010.3 ± 1175.5	0.17 ± 0.06	1601.7 ± 416.6	1665.8 ± 513.9	0.80 ± 0.61	1.62 ± 1.43

*Midazolam*						
Group A	755.7 ± 172.1	0.25 ± 0.04	416.8 ± 82.3	424.9 ± 77.9	1.51 ± 1.14	1.90 ± 1.63
Group B	585.6 ± 38.3	0.24 ± 0.07	283.7 ± 65.2^*∗∗*^	292.0 ± 75.1^*∗∗*^	0.92 ± 0.63	1.17 ± 1.00
Group C	650.9 ± 217.9	0.25 ± 0.05	295.7 ± 62.7^*∗∗*^	299.5 ± 60.0^*∗∗*^	0.72 ± 0.22	0.91 ± 0.57

*Dextromethorphan*						
Group A	342.7 ± 114.9	0.23 ± 0.20	540.7 ± 119.7	595.3 ± 122.2	3.12 ± 1.70	5.57 ± 3.52
Group B	428.8 ± 52.0	0.12 ± 0.12	760.6 ± 184.9^*∗*^	788.7 ± 211.0	3.66 ± 1.34	4.64 ± 2.04
Group C	464.2 ± 69.8^*∗*^	0.34 ± 0.28	734.3 ± 118.5^*∗*^	757.2 ± 105.4^*∗*^	3.18 ± 1.03	4.10 ± 2.11

*Phenacetin*						
Group A	3458.3 ± 708.8	0.31 ± 0.07	1886.7 ± 602.0	1944.1 ± 578.1	0.89 ± 0.49	1.45 ± 1.16
Group B	4484.6 ± 366.7	0.26 ± 0.08	2025.4 ± 512.1	2067.7 ± 509.9	0.55 ± 0.32	0.74 ± 0.73
Group C	4745.9 ± 1238.9	0.24 ± 0.04	1922.8 ± 595.2	1943.5 ± 581.4	0.62 ± 0.52	1.01 ± 0.92

*Tolbutamide*						
Group A	12402.5 ± 2375.8	0.06 ± 0.03	122412.8 ± 25958.5	141826.4 ± 36570.0	7.89 ± 0.72	11.73 ± 2.93
Group B	12397.5 ± 920.5	0.07 ± 0.01	124685.2 ± 19824.0	164218.1 ± 44237.0	8.75 ± 0.69	16.55 ± 5.52
Group C	13146.1 ± 3591.0	0.11 ± 0.10	112713.7 ± 19708.0	148136.8 ± 40794.2	8.31 ± 1.22	16.69 ± 13.26

^*∗*^ *P* < 0.05, different from group A; ^*∗∗*^*P* < 0.01, significantly different from group A.

**Table 4:** The pharmacokinetic parameters of the analytes (*n* = 6).

Analytes and group	*C*_max_ (ng/mL)	*T*_1/2_ (h)	*T*_max_ (h)	Ke (h^−1^)	AUC_(0–tn)_ (h·ng/mL)	AUC_(0–∞)_ (h·ng/mL)
*Metoprolol tartrate*						
Group A′	419 ± 181	1.28 ± 0.45	0.19 ± 0.06	0.62 ± 0.27	454 ± 113	529 ± 243
Group B′	307 ± 240	1.71 ± 1.11	0.38 ± 0.37	0.71 ± 0.65	350 ± 130	357 ± 136^*∗*^
Group C′	220 ± 81^*∗*^	1.73 ± 0.97	0.52 ± 0.49	0.54 ± 0.33	280 ± 69^*∗∗*^	285 ± 70^*∗∗*^

*O-demethylmetoprolol*						
Group A′	157 ± 42	2.06 ± 0.61	0.73 ± 0.59	0.37 ± 0.12	456 ± 91	473 ± 126
Group B′	122 ± 28	2.56 ± 0.92	1.31 ± 0.37^*∗*^	0.30 ± 0.11	411 ± 49	414 ± 49
Group C′	160 ± 55	2.16 ± 0.84	1.21 ± 0.67	0.38 ± 0.18	536 ± 145^*∗*^	540 ± 146^*∗*^

*α-Hydroxymetoprolol*						
Group A′	389 ± 159	2.08 ± 0.53	0.22 ± 0.04	0.36 ± 0.10	882 ± 144	893 ± 148
Group B′	238 ± 90^*∗*^	3.02 ± 1.32	1.31 ± 0.37^*∗∗*^	0.27 ± 0.12	736 ± 163	765 ± 151
Group C′	377 ± 102	2.26 ± 0.88	0.97 ± 0.69^*∗*^	0.35 ± 0.13	997 ± 162	1003 ± 162

^*∗*^ *P* < 0.05, different from group A′; ^*∗∗*^*P* < 0.01, significantly different from group A′.

## Data Availability

The data used to support the findings of this study are available from the corresponding author upon request.
